# Supplementary figures and images for: Magnitude and Kinetics of T Cell and Antibody Responses During H1N1pdm09 Infection in Inbred Babraham Pigs and Outbred Pigs
Source: Front Immunol. 2021 Feb 2;11:604913. doi: 10.3389/fimmu.2020.604913 (PMC7884753; doi:10.3389/fimmu.2020.604913)

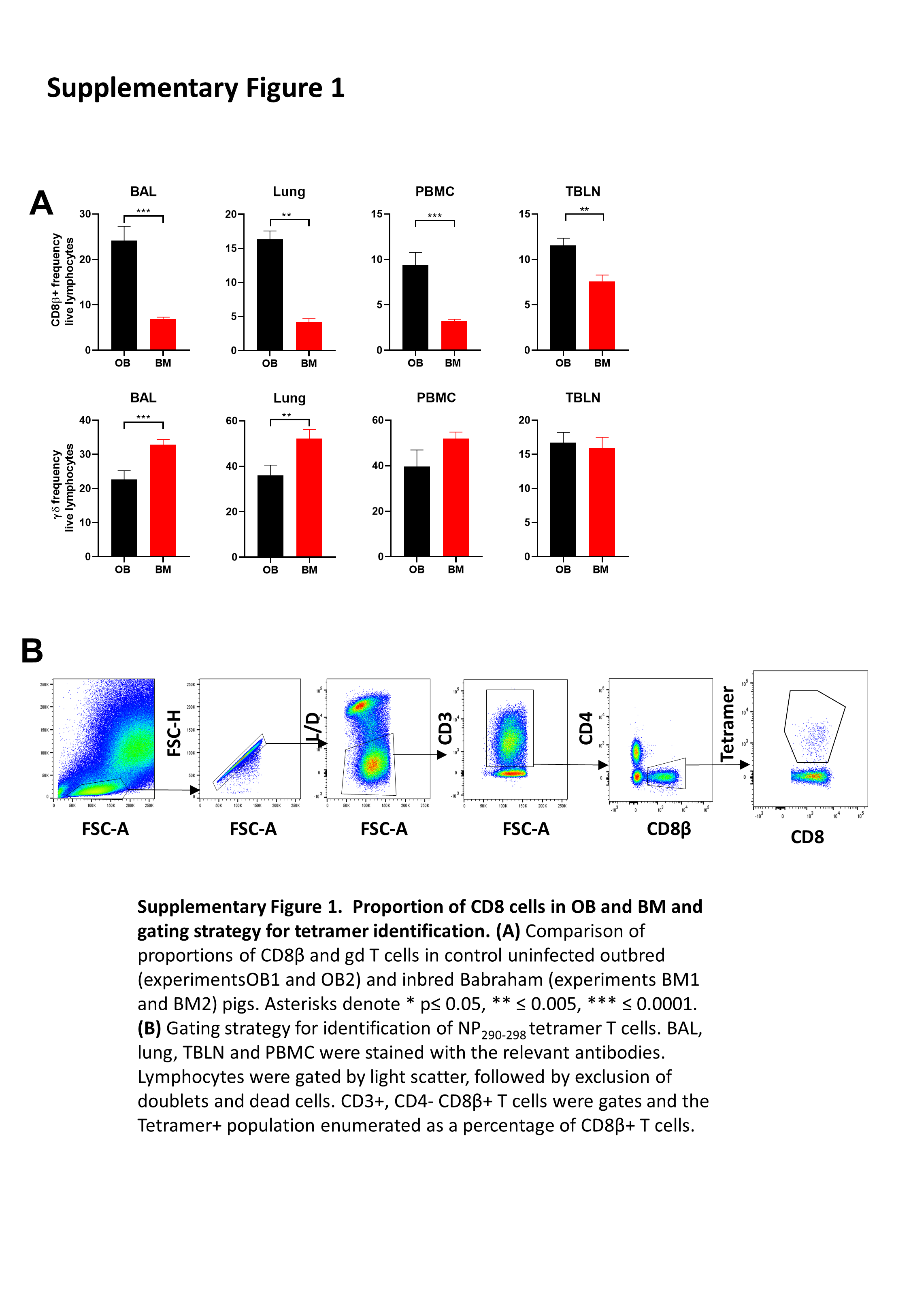

Supplement: Supplementary file 2 [file Image_1.tif]

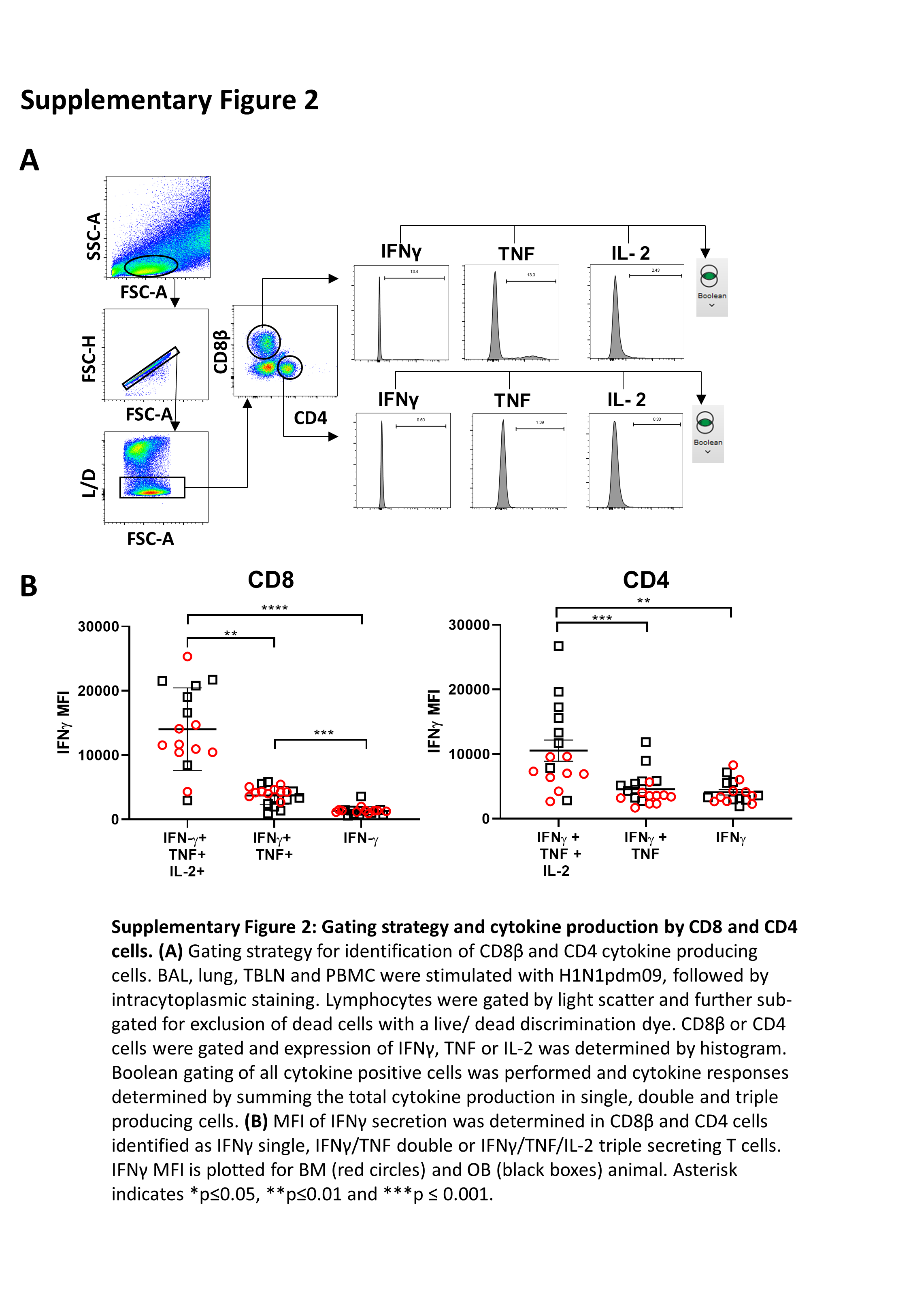

Supplement: Supplementary file 3 [file Image_2.tif]

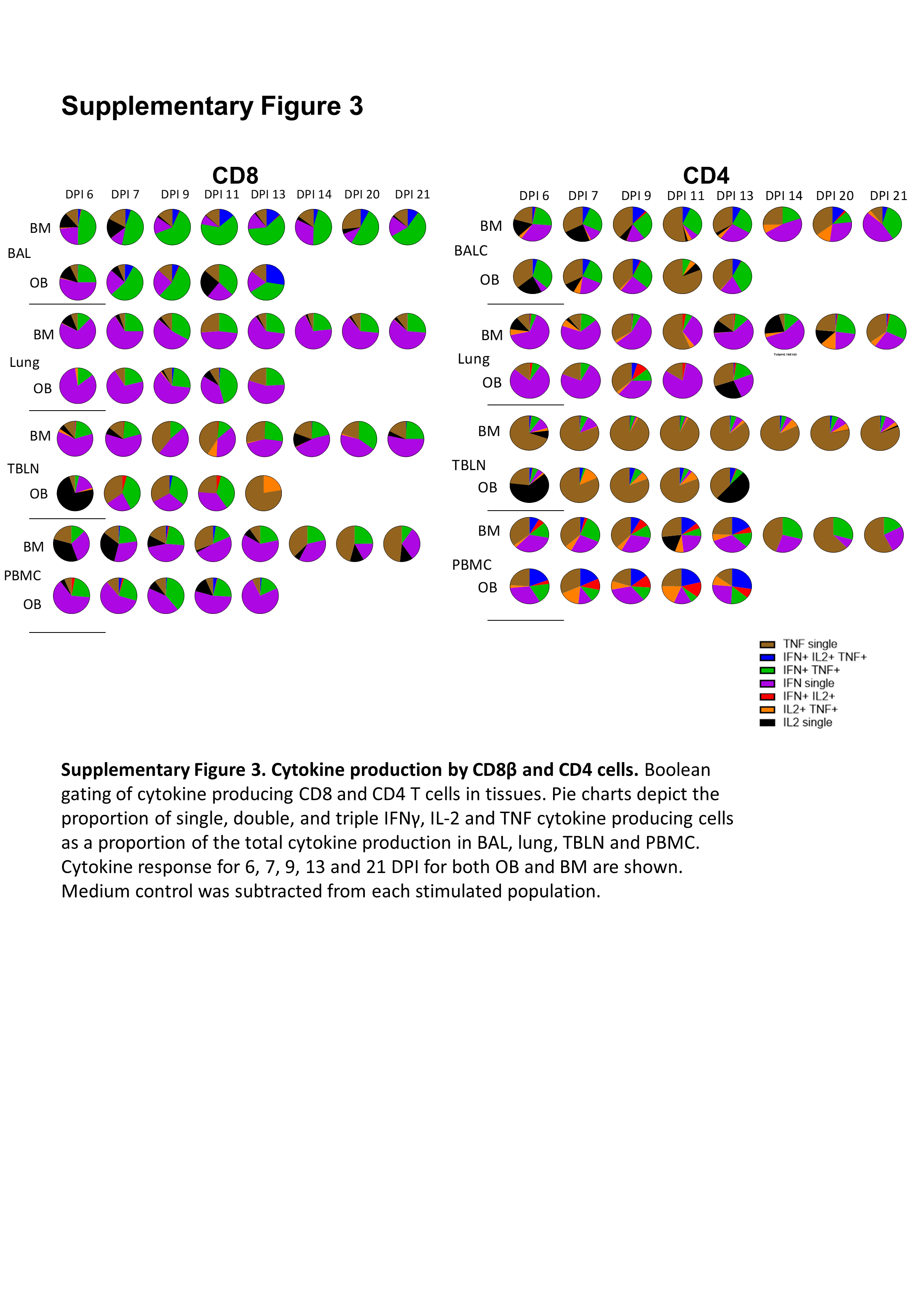

Supplement: Supplementary file 4 [file Image_3.tif]

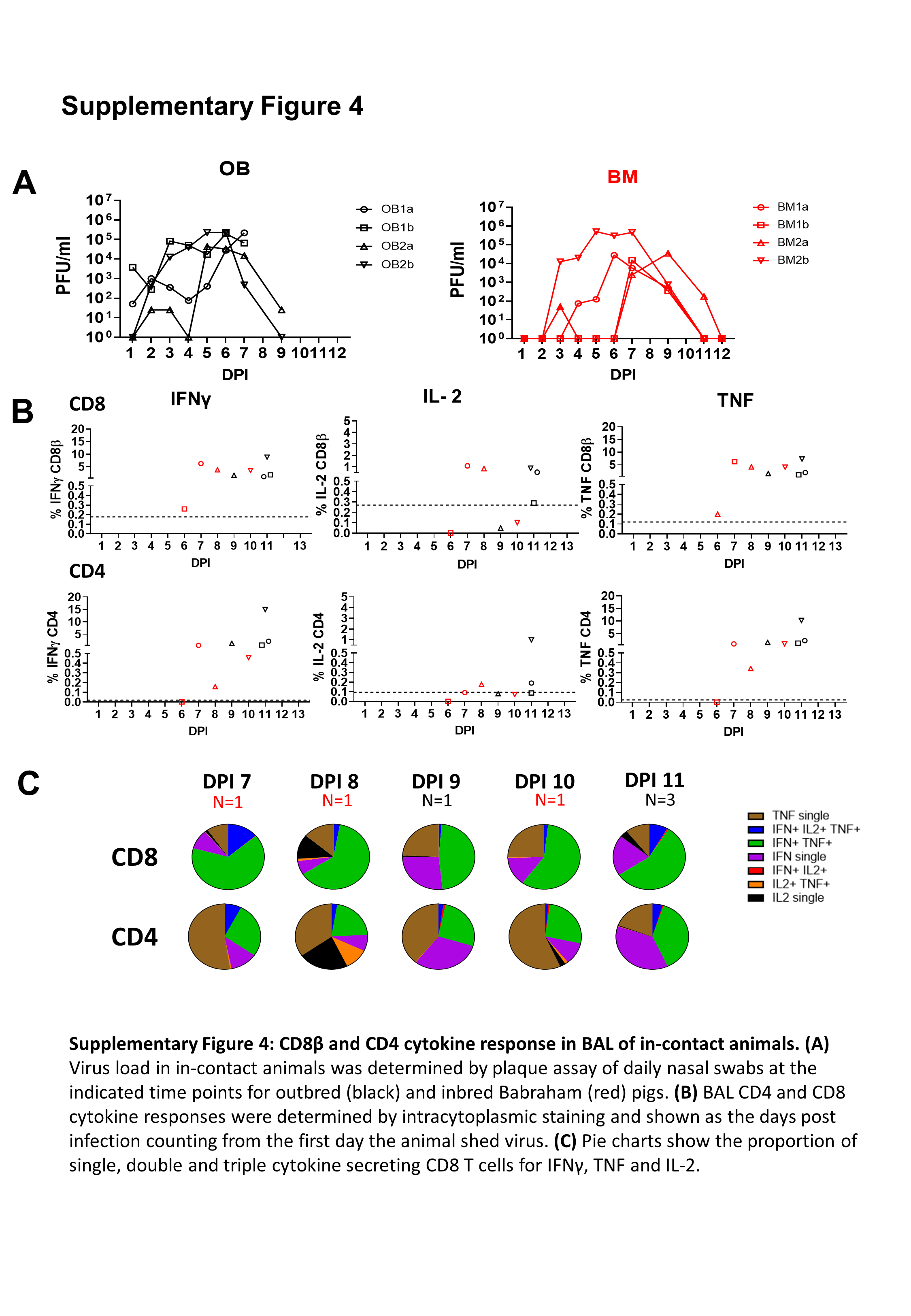

Supplement: Supplementary file 5 [file Image_4.tif]

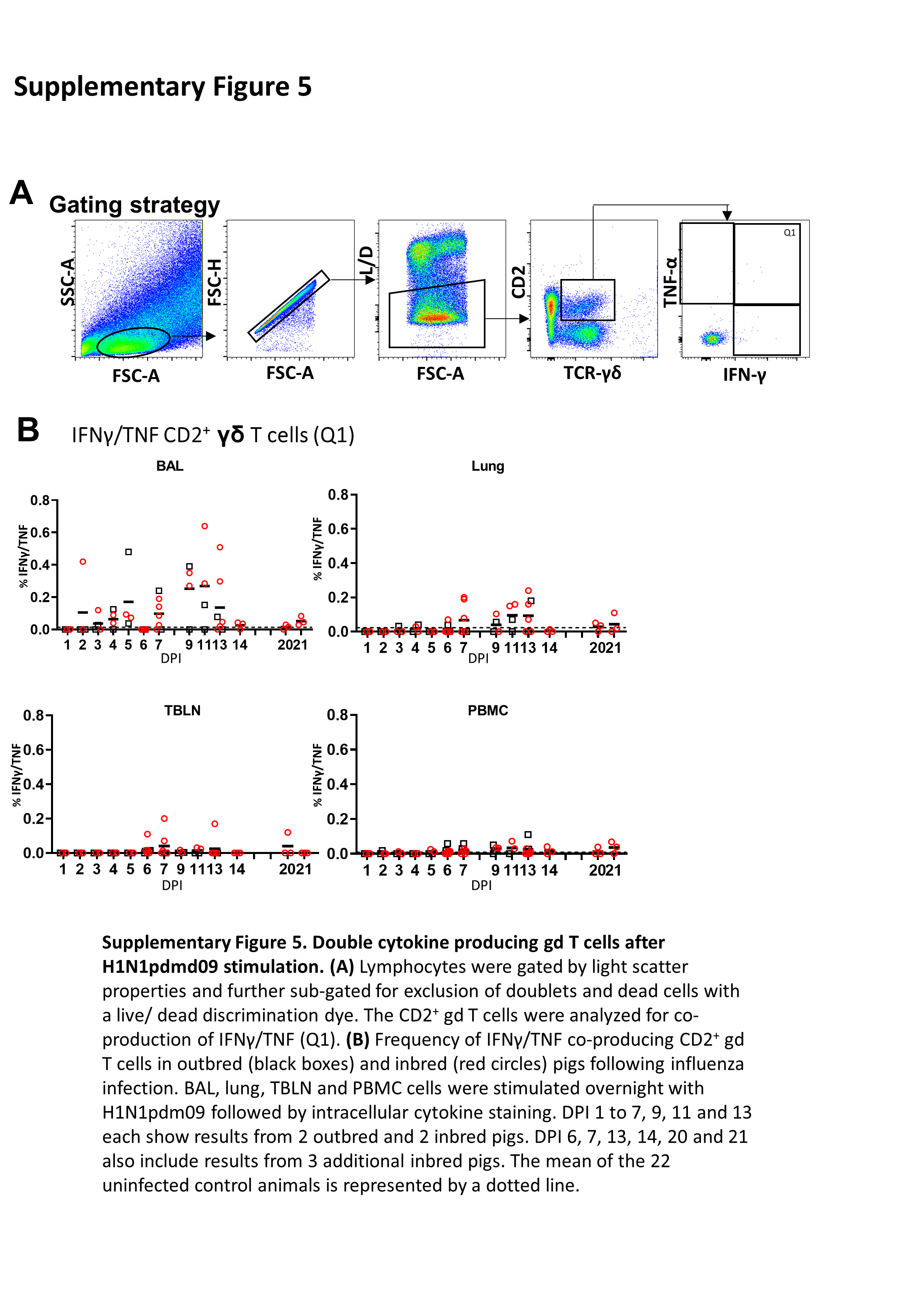

Supplement: Supplementary file 6 [file Image_5.tif]

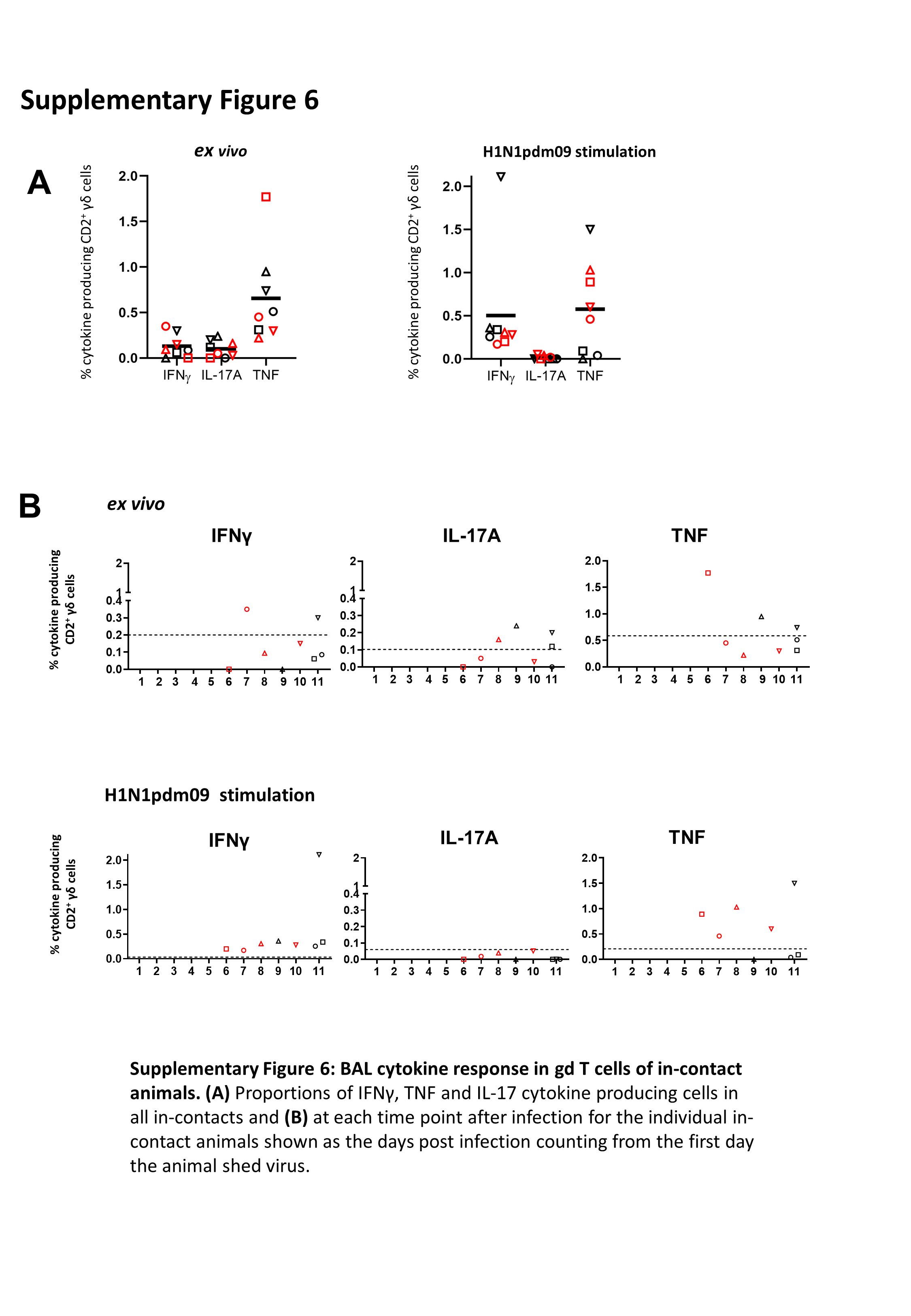

Supplement: Supplementary file 7 [file Image_6.tif]

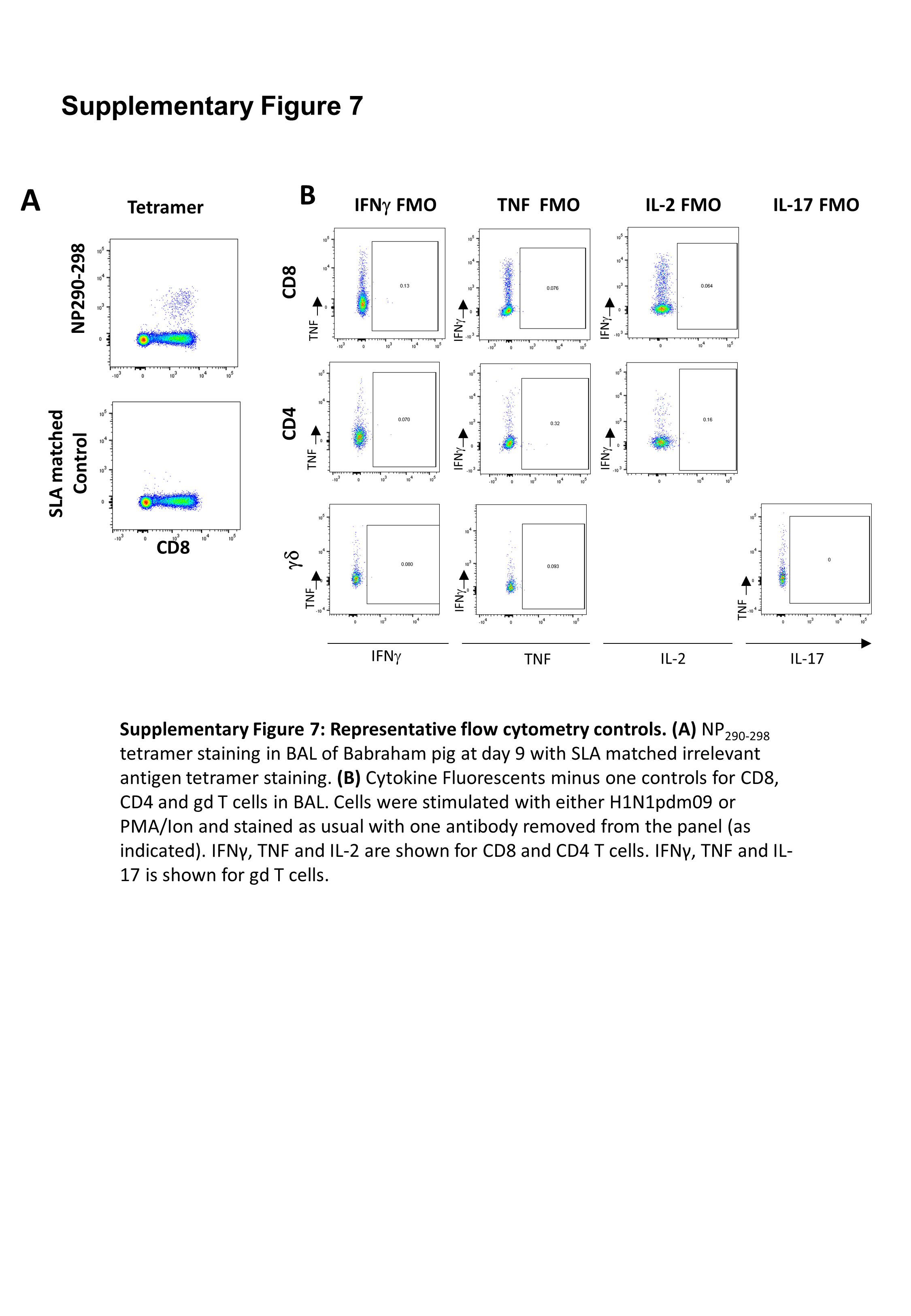

Supplement: Supplementary file 8 [file Image_7.tif]
