# Supplementary material for: Magnitude and Kinetics of T Cell and Antibody Responses During H1N1pdm09 Infection in Inbred Babraham Pigs and Outbred Pigs
Source: Front Immunol. 2021 Feb 2;11:604913. doi: 10.3389/fimmu.2020.604913 (PMC7884753; doi:10.3389/fimmu.2020.604913)
Supplement: Supplementary file 9 [file Table_1.docx]

**Supplementary Table 1.** Estimated parameters (95% confidence intervals) for logistic growth curves used to model the kinetics of antibody responses in inbred Barbraham and outbred pigs following infection with H1N1pdm09 swine influenza virus.

| **antibody response*** | **upper asymptote (log_10_ titre)** | | **rate of increase in titre (per day)** | | **time of maximum increase (dpi)** | |
| --- | --- | --- | --- | --- | --- | --- |
|  | **Babraham** | **outbred** | **Babraham** | **outbred** | **Babraham** | **outbred** |
| **serum IgG H1N1pdm** | 4.0 (3.7, 4.3) | 3.7 (3.2, 4.2) | 1.1 (0.5, 1.6) | 2.0 (0.4, 3.6) | 7.1 (6.6, 7.6) | 6.7 (6.3, 7.2) |
| **BAL IgG H1N1pdm** | 3.2 (2.9, 3.6) | 3.8 (2.8, 4.7) | 0.5 (0.3, 0.7) | 0.8 (0.3, 1.1) | 9.5 (8.5, 10.5) | 9.2 (8.0, 10.5) |
| **serum IgA H1N1pdm** | 2.1 (1.8, 2.4)† | 2.7 (2.2, 3.2)† | 2.0 (0.0, 4.1) | 1.8 (0.0, 3.5) | 6.1 (5.6, 6.5) | 6.2 (5.6, 6.8) |
| **BAL IgA H1N1pdm** | 3.2 (2.9, 3.5) | 3.7 (2.8, 4.5) | 0.6 (0.4, 0.8) | 0.9 (0.4, 1.4) | 9.0 (8.1, 9.9) | 9.2 (8.3, 10.2) |
| **serum IgG pH1** | 2.5 (2.1, 2.8) | 3.0 (2.5, 3.6) | 1.1 (0.1, 2.0) | 2.0 (0.0. 4.1) | 6.6 (5.9, 7.4) | 6.6 (6.0, 7.2) |
| **BAL IgG pH1** | -‡ | -‡ | -‡ | -‡ | -‡ | -‡ |
| **serum IgA pH1** | 2.2 (2.0, 2.3) | 2.4 (2.1, 2.7) | 3.0 (1.5, 4.6) | 3.4 (0.2, 6.6) | 6.7 (6.4, 6.9) | 6.3 (5.9, 6.6) |
| **BAL IgA pH1** | 1.6 (1.3, 1.9) | 1.9 (1.2, 2.6) | 0.7 (0.0, 1.3) | 0.9 (0.0, 2.0) | 8.0 (6.2, 9.8) | 8.4 (6.5, 10.2) |
| **serum MN** | 2.0 (1.8, 2.2) | 2.2 (1.8, 2.5) | 1.6 (0.4, 2.7) | 1.4 (0.3, 2.5) | 6.1 (5.7, 6.6) | 5.9 (5.2, 6.6) |
| **serum HAI** | 2.5 (2.3, 2.6) | 2.5 (2.3, 2.8) | 1.3 (0.8, 1.8) | 1.3 (0.7, 1.9) | 5.5 (5.2, 5.8) | 5.6 (5.1, 6.0) |
| **serum ELLA** | 1.7 (1.6, 1.8) | 1.9 (1.7, 2.2) | 2.6 (1.2, 4.1) | 1.8 (0.5, 3.2) | 6.4 (6.1, 6.7) | 6.2 (5.7, 6.6) |
| **BAL MN** | 1.4 (1.0, 1.8) | -‡ | 0.3 (0.2, 0.5) | -‡ | 10.8 (8.2, 13.3) | -‡ |
| **BAL HAI** | -‡ | -‡ | -‡ | -‡ | -‡ | -‡ |
| **BAL ELLA** | 0.7 (0.4, 0.9) | 1.4 (0.6, 2.3) | 0.8 (0.0, 2.0) | 0.7 (0.0, 1.5) | 9.1 (6.0, 12.2) | 9.4 (6.4, 12.4) |

* BAL - broncho-alveolar lavage

† estimates for Babraham and outbred pigs differ significantly (P=0.05)

‡ curves could not be fitted to the data (model would not converge)
